# Soil erosion and hydroclimatic hazards in major African port cities: the case study of Tangier

**DOI:** 10.1038/s41598-023-40135-3

**Published:** 2023-08-12

**Authors:** Adil Salhi, Yassin El Hasnaoui, Pedro Pérez Cutillas, Essam Heggy

**Affiliations:** 1 Geography and Development Group, Abdelmalek Essaadi University, FLSH, Martil, Morocco; 2 Department of Geography, University of Murcia, C. Santo Cristo, 1, 30001 Murcia, Spain; 3 NASA Jet Propulsion Laboratory, California Institute of Technology, 4800 Oak Grove Drive, Pasadena, CA 91109 USA; 4 Viterbi School of Engineering, Powell Hall of Engineering, University of Southern California (USC), 3737 Watt Way, Los Angeles, CA 90089 USA

**Keywords:** Hydrology, Environmental sciences, Natural hazards

## Abstract

Land degradation and soil erosion are becoming increasingly problematic in Africa's rapidly developing urban areas, particularly in Major Port Cities. Uncontrolled expansion and human pressures are hindering planning, adaptation, and conservation efforts. To understand the extent of these issues, this study combined morphometric analysis, soil loss calculation, field monitoring, and remote sensing and GIS tools to assess soil erosion in the Metropolis of Tangier (Morocco) located at the confluence of the Mediterranean Sea and the Atlantic Ocean at the Strait of Gibraltar. The study relied on data from 13 rain gauge stations, official reports, and remote sensing acquisitions, as well as field observations. Results showed an average soil erosion rate of 24.2 t/ha/year, equivalent to an annual soil loss of 588,051 t/year. This high rate was largely due to areas with a high erosion risk (99.8%), covering only 8.3% of the territory, which were characterized by recently burned topsoil, fallow land, and steep slopes. These areas included both uncontrolled neighbourhoods and areas for planned urban and industrial expansion, posing a threat to the landscape's sustainability and socio-economic prospects. The morphometric analysis revealed its high vulnerability to erosion and degradation, with the highest soil loss rates observed in the eastern and western regions. The study also found that flash floods caused by hydroclimatic hazards can lead to significant damage to infrastructure and equipment, particularly in western sub-basins and mountainous regions. In conclusion, the use of remote sensing and GIS technologies provided valuable insights into the physical characteristics and vulnerability of the Tangier Metropolis to land degradation and soil erosion. These findings emphasize the need for effective land management practices and conservation measures to mitigate the impacts of land degradation and soil erosion in the face of climate change. This information is crucial for decision-makers and stakeholders to develop strategies to address these pressing issues.

## Introduction

Land degradation is a global environmental challenge that severely impacts on food and energy security, declining living standards, and biodiversity loss^1,2^. The latter is driven by multiple anthropogenic activities, and climate drivers^3^. It is an increasingly concerning issue, particularly in fast-growing emerging cities, where rapid urbanization and uncontrolled expansion have resulted in significant alterations to hydrologic processes and negative socioecological implications^4,5^.

The relationship between climate change, hydrology, and land degradation is complex and interrelated. Climate change affects hydrologic processes, which in turn can contribute to land degradation^6,7^. Conversely, land degradation can also increase the vulnerability of a region to the impacts of climate change^6,8^. To address this challenge effectively, it is crucial to identify the drivers of degradation at the appropriate spatial scale and use a range of tools and techniques such as geographic information systems and remote sensing to assess changes in hydrologic processes^3,9^.

In the context of port cities, land degradation poses a significant environmental issue, adversely affecting ecosystems and jeopardizing the long-term sustainability of urban development^10^. The rapid expansion of port cities and inefficient transformation projects contributes to land degradation, leading to the depletion of fertile soils and increased sedimentation in neighboring hydrologic systems^11^. Additionally, the combination of intensive land use practices and the modification of natural drainage patterns and waterways due to the intensification of anthropogenic activity in the hinterland of port cities accelerates soil erosion, which thereby threatening the long-term viability of ecosystems^12^.

Considering the rising adverse impacts of climate change on port cities, it becomes essential to understand their vulnerabilities and explore potential adaptation strategies. These vulnerabilities encompass not only the impacts of climate change but also public perception, inadequate infrastructure, and limited adaptive capacity^13,14^.

The Tangier Metropolis is treated herein as a case study for the above challenges for several reasons. Firstly, the city and its surroundings have experienced significant changes in its land use, including uncontrolled urban expansion and intensive industrial activity, which have extensively impacted soil erosion and pose a significant environmental concern^15^. Secondly, the area's challenges result from rapid urban growth, erosion-prone landforms, and increased climate fluctuation^16,17^. Thirdly, the study of Tangier serves as a crucial case for understanding the mechanisms of land degradation in growing urban areas and the interplay between hydrological and anthropogenic pressures^4^.

The port of Tangier is one of the busiest in Africa, handling approximately 3 million containers per year^18^. Most of these containers are destined for Europe and other parts of the world, making Tangier an essential hub for global trade. However, the recent soil erosion in the region threatens to undermine the city's role as a critical player in the global economy, potentially leading to significant economic losses. Therefore, understanding the causes and consequences of soil erosion in Tangier is paramount. The issue of soil erosion in port cities is not unique to Tangier. Other cities in the region, such as Alexandria, Tunis and Algiers, are also facing similar challenges^19,20^. These cities share common vulnerabilities, including climate fluctuations and inadequate land-use planning, and inefficient transformation projects of their waterscape. Therefore, the findings of this investigation have significant implications for other port cities in the Mediterranean and North Africa.

Therefore, to comprehensively assess of the causes and consequences of land degradation, we employ a combination of advanced modeling techniques and in-situ monitoring in order to quantify erosion drivers and the best practices for their prevention and mitigation.

This study seeks to achieve the following specific objectives: (1) fill critical knowledge gaps in understanding the causes and consequences of land degradation in the Tangier Metropolis and similar cases, (2) provide insights and recommendations for improving land use management practices in the study area and beyond, based on a comprehensive assessment of erosion factors and best practices for prevention and mitigation. (3) Highlight effective strategies and interventions for preventing and mitigating land degradation.

The anticipated outcomes of this study hold significant implications for both the sustainable development of the study area and the broader global efforts to address land degradation as an environmental challenge. By generating a comprehensive understanding of the driving factors behind land degradation in fast-growing emerging cities, the research will inform improved planning and management of land use promoting sustainable practices and contributing to the global endeavor of addressing this pressing environmental issue.

## Methods

### Study area

**Figure 1:**
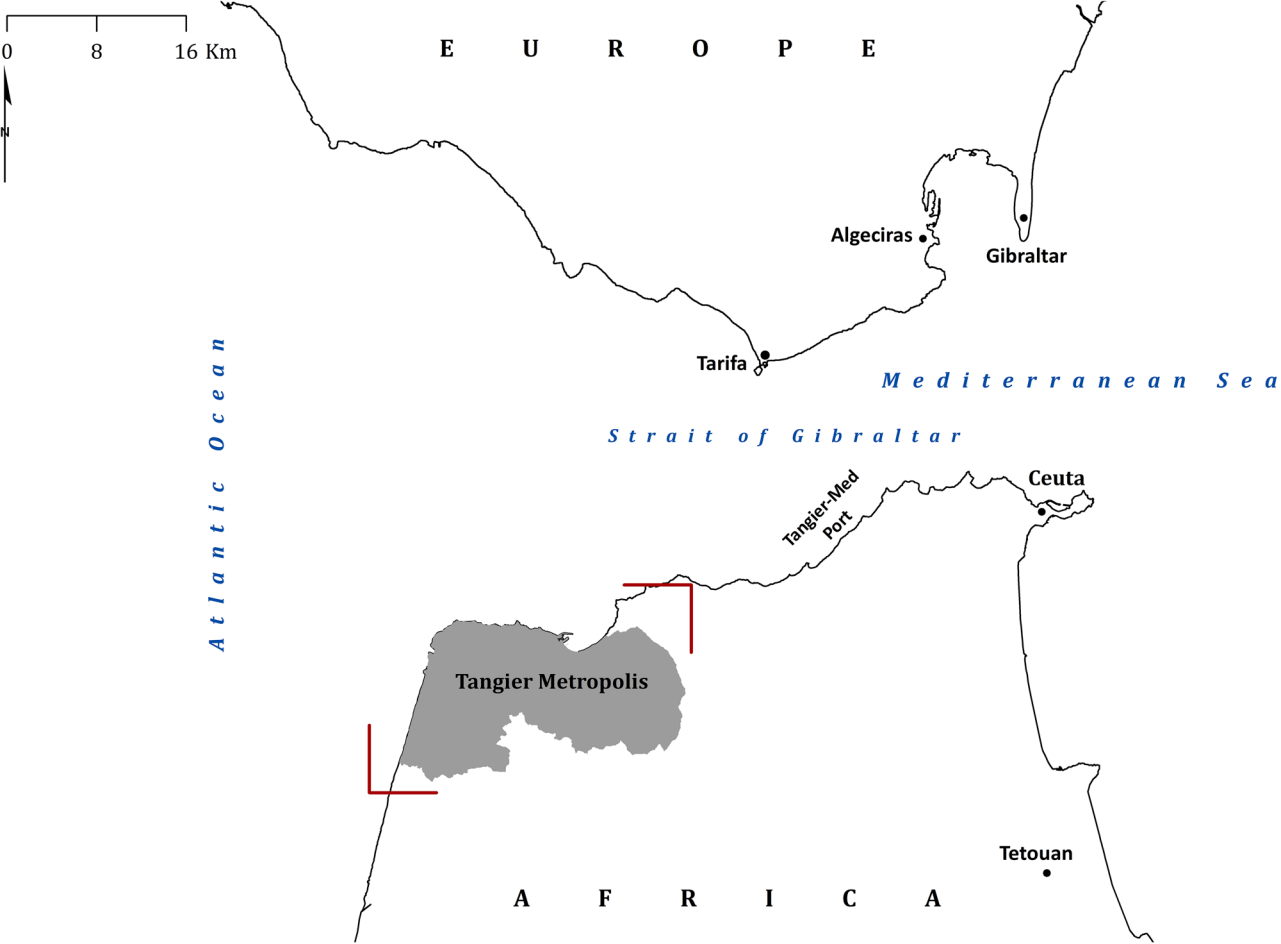
Location of the study area (in red brackets) (this figure was generated using ArcGIS 10.6 https://desktop.arcgis.com/en/arcmap/10.6/get-started/installation-guide/installing-on-your-computer.htm).

The study area is the metropolis of Tangier (Morocco) located at the confluence of the Mediterranean Sea and the Atlantic Ocean at the Strait of Gibraltar (Fig. 1). In addition to the Mediterranean and the Atlantic bordering the study area to the north and west respectively, this major port city is limited to the south by the mountain range of Dar Z’hiro, and by the Fahs plateaus to the east. As the main gateway between Africa and Europe, it is undergoing continuous socioeconomic transformation, with three highly active and two programmed industrial zones and rapid demographic growth in the past 2 decades.

It is situated along the southern shoreline of the Strait of Gibraltar, a major global shipping route. Tangier port city encompasses a triangular region with distinct features: an extensive port complex to the east, a sprawling metropolis to the west, an industrial multinational, and an ongoing city construction site towards the south^4^. Significant efforts have been directed towards developing various sectors in this port city. This comprehensive endeavor has prioritized the establishment of industrial, commercial, and economic zones, all of which are aligned with a comprehensive national vision. Two important ports are present. The first is the Tangier city port, located in the center of the metropolis. Over time, this port has significantly reduced its activity and transformed into a marina, following the establishment of the second port. The latter is Tangier-Med, situated approximately 43 km to the east. This strategic port holds immense importance for Morocco's economic and social development; it stands as the largest port in terms of capacity in the Mediterranean and Africa, with the ability to handle up to 9 million containers. Functioning as a global logistics gateway, the port serves as a thriving industrial hub, accommodating over 1100 companies across various automotive, aeronautics, logistics, textiles, and trade sectors. Together, these businesses contribute to an annual export turnover surpassing 5.9 billion USD.

From the hydrological point of view, it is divided between eight sub-basins drained by watercourses bearing the same names. Five of them flow towards the Mediterranean (Msaben, Mlaleh, Mghougha, Souani, and Lihoud), two towards the Atlantic (Boukhalef and Gueznaia), and one (Spartel-Achkkar) towards the two seaboards.

The area is surrounded by the mountainous terrain of Al-Fahs to the east, Jbel Kebir to the northwest, and Dar Z’hiro to the south. The Boukhalef plain dominates the West, while the Mghougha basin and some narrow intra-hill depressions dominate the center. The rest of the area is made up of scattered plateaus (Marshan and Beni Makada) and the hills of Al-Sharf, Al-Jebilat, and Sidi Kassem. This geomorphology results in a relief divided halfway between weak and steep slopes.

Tangier has seen rapid demographic growth due to both local village migration and immigration from outside the region, attracted by the city's strong economic appeal associated with the growth of industry and tourism^15,21^. As a result, the population of the metropolis reached over 1.3 million people in 2022. The metropolis has also been characterized by a high rate of industrialization, with the establishment of numerous industrial zones and facilities. In terms of tourism, Tangier has a rich history and cultural heritage, as well as a scenic location, attracting millions of tourists each year^22^.

The climate in Tangier is Mediterranean, with mild and wet winters and warm, dry summers^4,23,24^. The average temperature ranges between 13 and 26 °C, with the highest temperatures typically occurring in July and August^25,26^. The region receives most of its precipitation in the winter months, with an average annual precipitation of 600 mm^27^.

### Morphometric analysis

The morphometric study was an important component of the research to understand the relationship between land degradation and hydrologic processes in the study area. The study aimed to quantify and analyze the physical characteristics of the region's landforms, including the relief, slope, aspect, and drainage patterns.

This study was performed using a combination of field observations and digital mapping techniques, including the use of the Shuttle Radar Topography Mission (SRTM) v3 digital elevation model (DEM) and ArcGIS software. The SRTM V3 DEM have a resolution of 30 m and provide an accurate representation of the sub-basin's elevation and slope.

In the analysis, various physical characteristics of the landforms in the Tangier Metropolis were analyzed, including form, topological, and drainage characteristics.

Form characteristics were calculated to describe the shape of the landforms, and included the following parameters:

- Area (A): the total surface area of a sub-basin.
- Length (L): the maximum length of a sub-basin.
- Width (W): the maximum width of a sub-basin^28^.
- Perimeter (P): the total length of the boundary of a sub-basin.
- Elongation ratio (ER): the ratio of the length to the width of a sub-basin, calculated as L/W^29^.
- Circularity ratio (CR): a measure of the circularity of a sub-basin, calculated as 4πA/P^2^^30^.
- Compactness coefficient (CC): a measure of how compact a sub-basin is, calculated as A/LW^31^.
- Form factor (FF): a measure of the deviation of a sub-basin from a perfect circle, calculated as P^2^/4πA^32^.

Topological characteristics were calculated to describe the elevation of the landforms and their relief, and included the following parameters:

- Max altitude (MA): the highest elevation of a sub-basin.
- Min altitude (MI): the lowest elevation of a sub-basin.
- Relief ratio (RR): the ratio of the difference between the max and min altitude of a sub-basin to the length of the sub-basin, calculated as (MA − MI)/L^33^.
- Relative relief (RRel): the ratio of the difference between the max and min altitude of a sub-basin to its mean altitude, calculated as (MA − MI)/((MA + MI)/2)^29^.
- Ruggedness number (RN): a measure of the roughness of a sub-basin, calculated as the standard deviation of the elevations^31^.
- Texture ratio (TR): the ratio of the ruggedness number to the mean elevation of the sub-basin, calculated as RN/(MA + MI)/2^34^.
- Hypsometric integral (HI): a measure of the distribution of elevations, calculated as the ratio of the total area lying above a certain elevation to the total area of the sub-basin^35^.

Drainage characteristics were calculated to describe the drainage patterns and water flow in the landforms, and included the following parameters:

- Stream length (SL): the total length of all streams in a sub-basin.
- Number of streams (NS): the total number of streams in a sub-basin.
- Drainage density (DD): the number of streams per unit area, calculated as NS/A^34^.
- Stream frequency (SF): the number of streams per unit length, calculated as NS/L^34^.
- Constant of channel maintenance (CCM): a measure of the balance between erosion and deposition in a stream channel, calculated as the slope of a stream multiplied by its drainage area^36^.
- Drainage intensity (DI): a measure of the amount of water flowing in a sub-basin, calculated as the ratio of the total discharge to the total drainage area^31^.
- Infiltration number (IN): a measure of the ability of the soil to absorb water, calculated as the ratio of the total infiltration to the total drainage area.

The morphometric study intended to provide valuable insights into the physical characteristics of the sub-basins in the Tangier Metropolis and to identify areas that are more prone to erosion and degradation. The results were used in conjunction with the results of soil loss modeling and field monitoring of soil erosion to providing a comprehensive understanding of the factors driving land degradation in the area.

### Soil loss modeling

In this study, the Revised Universal Soil Loss Equation (RUSLE) was used to assess soil erosion. The RUSLE model was developed by the USDA Natural Resources Conservation Service (NRCS) and is based on the following five factors: rainfall erosivity (R), soil erodibility (K), slope length and gradient (LS), cover and management (C), and support practices (P)^37^. The model uses these factors to calculate the average annual soil loss (A) (t/ha/year) according to Eq. (1)^37^.

1 $$\mathrm{A}=\mathrm{R}\cdot \mathrm{K}\cdot \mathrm{LS}\cdot \mathrm{C}\cdot \mathrm{P}$$

To validate the findings, ground validations observations were carried out within the study area to assess soil erosion. These field observations (direct visual inspection, measurement of erosion features, sediment deposition analysis, erosion indicator assessments, and data integration and analysis), which are detailed in the subsequent subsection, were then compared to the predictions generated by the RUSLE model. The outcomes of the field observations played a crucial role in assessing the accuracy of the model's soil erosion predictions and identifying any potential sources of error.

The data comes from 13 rainfall stations in and adjacent to the study area which are well described in two recent papers^25,27^, reports from the geological service of Morocco, the soil fertility map of Morocco (Fertimap) produced by the National Institute for Agronomic Research (INRA), and the analysis of remote sensing data (SRTM V3 and the annual average of Landsat 8 acquisitions from 2020,which were processed using Google Earth Engine, with a spatial resolution of 30 m).

R-factor is the susceptibility to cause erosion by precipitation and runoff which can lead to the uprooting of small parts of soil. It depends on the dynamic energy of precipitation, its frequency, and its duration. It was assessed according to a mathematical formula adapted to the Moroccan settings and the available data^38^ as shown in Eq. (2):

2 $$\mathrm{R}=143\cdot \mathrm{log}\left(\mathrm{P}\cdot {\mathrm{P}}_{\mathrm{i}}^{2}\cdot {10}^{-6}\right)+89.7$$

where P is the mean annual precipitation, and P_i_ is the average maximum precipitation in 24 h.

Many estimates have been developed to approximate soil erodibility from readily available soil properties and standard profile descriptions. Here, K-factor values for soils of different textural classes were taken from a recent estimate^39^ as shown in Table 1.

**Table 1:** K-factor value according to soil texture (Benavidez et al.^39^).

Soil texture	K-factor
Sand	0.05
Loamy sand	0.07
Sandy loam	0.23
Silt	0.35
Loam	0.25
Sandy clayey loam	0.18
Silty loam	0.30
Clay	0.20
Silty clay	0.19
Sandy clay	0.09
Clayey loam	0.22
Silty clayey loam	0.28

The LS factor considers the effect of topography on erosion. It is calculated from SRTM V3 digital elevation model based on S (slope steepness) and L (slope length) factors according to Eq. (3)^40^:

3 $$\mathrm{LS}=\sqrt{\frac{\mathrm{L}}{22}(0.065+0.45\mathrm{S}+{0.0065\mathrm{S}}^{2} )}$$

C-factor values were attributed to the land use and land cover (LULC) map, extracted from the Esri LULC layer derived from Sentinel-2 imagery of 2020 at 10m spatial resolution. High values are assigned for no vegetation cover and low values when vegetation or crop was available, as well-described in the literature^41,42^.

An overall P-factor was assessed for individual support practices used to reduce erosion. The P value is 1 for soils without support practices and close to zero when proper erosion control measures are implemented^43^. From the supervised classification of the Sentinel-2 imagery of 2020 supported by field missions for verification, the values of the P-factor have been assigned to the different land uses (0.8 for sparse vegetation and 1 for built-up, agriculture, and water bodies^44^).

The various data inputs were processed in ArcGIS to create the five thematic maps. These were multiplied using the raster calculator tool according to the RUSLE relationship to generate the composite map of estimated erosion loss.

### Field monitoring

The field monitoring component aimed to gather valuable data on soil erosion to complement the results obtained from the RUSLE modeling. It involved a comprehensive set of field observations (direct visual inspection, measurement of erosion features, sediment deposition analysis, erosion indicator assessments, and data integration and analysis) conducted within specific locations in the study area. To ensure accuracy, a combination of established field observation techniques and measurements was employed, including direct visual inspection and measurements of erosion features and sediment deposition levels. The duration of the field observations spanned approximately 12 months (2021–2022), ensuring a representative spatial coverage of the study area, and capturing seasonal variations. The locations for the field observations were carefully selected to cover different land use types and topographic conditions. We focused on areas susceptible to erosion, such as steep slopes, agricultural fields, and areas with significant human activities that could contribute to erosion processes.

The field monitoring process included the following steps:

- Direct visual inspection: a trained researcher conducted on-site inspections to visually assess the presence and severity of erosion features, including rills, gullies, and sediment deposition. These observations provided qualitative information on land degradation's extent and spatial distribution. The selection of erosion field features was complemented by satellite-based observations using the annual average of Sentinel-2 imagery acquisitions from 2020, which were processed using Google Earth Engine, at a spatial resolution of 10 m. The satellite imagery was processed using ArcGIS software to identify erosion and sediment deposition areas.
- Measurement of erosion features: on-the-ground measurements of erosion features, such as the dimensions of rills and gullies, were conducted using standard surveying techniques. This quantitative data enabled us to validate the physical characteristics of erosional processes.
- Sediment deposition analysis: the sediment deposition resulting from erosion was carefully analyzed to endorse erosion modelling.
- Erosion indicator assessments: various erosion indicators were assessed during the field observations, including vegetation cover, lithology, and surface roughness. These indicators allowed to evaluate the overall condition of the selected location and identify areas prone to erosion.
- Data integration and analysis: the satellite-based and the on-the-ground observations were integrated and analyzed to provide a comprehensive understanding of the causes and consequences of soil erosion.

The field monitoring provided valuable data to ratify the outcomes from the RUSLE modeling and to support the study’s overall goal, which was to provide a comprehensive assessment of the causes and consequences of land degradation in the study area.

## Results and discussion

### Physical characteristics and susceptibility of sub-basins to land degradation

The morphometric parameters of the sub-basins were analyzed to better understand their physical characteristics and their relationship with land degradation (Table 2). The findings indicate that the sub-basins are small, elongated, non-circular, and compact with irregular shapes. The topographical characteristics indicate that the sub-basins have a moderate relief and a medium to a steep slope, with flatter sub-basins, textured, and irregular short tributaries.

**Table 2:** Morphometric characteristics of the eight sub-basins of Tangier.

	Sub-basins								Recap
	Msaben	Mlaleh	Mghougha	Souani	Lihoud	Spartel-Achkkar	Boukhalef	Gueznaia	
Form characteristics									
Area (km^2^)	11.4	22.3	77.7	13.6	28.1	24.0	39.8	29.1	245.9
Length (km)	6.8	8.6	11.5	5.1	8.0	8.5	8.7	9.3	23.8
Width (km)	1.7	2.6	6.8	2.7	3.5	2.8	4.6	3.1	10.4
Perimeter (km)	17.2	25.4	49.0	30.3	32.0	35.3	37.8	30.6	99.3
Elongation ratio	0.6	0.6	0.9	0.8	0.8	0.7	0.8	0.7	0.7
Circularity ratio	0.5	0.4	0.4	0.2	0.3	0.2	0.4	0.4	0.3
Compactness coefficient	1.5	1.5	1.6	2.3	1.7	2.0	1.7	1.6	1.8
Form factor	0.2	0.3	0.6	0.5	0.4	0.3	0.5	0.3	0.4
Topological characteristics									
Max altitude (m)	427	478	464	102	270	330	263	252	478
Min altitude (m)	0	0	0	0	0	0	0	0	0
Relief ratio	62.4	55.7	40.5	20.0	34.0	38.8	30.4	27.0	20.1
Relative relief	24.8	18.9	9.5	3.4	8.4	9.4	7.0	8.2	4.8
Ruggedness number	0.1	0.1	0.0	0.0	0.0	0.1	0.0	0.0	0.0
Texture ratio	2.0	3.1	4.6	1.2	2.1	2.3	2.6	2.7	7.1
Hypsometric Integral	0.1	0.1	0.2	0.1	0.1	0.1	0.2	0.1	0.5
Drainage characteristics									
Stream length (km)	12.2	22.2	84.7	11.2	30.3	29.2	43.2	29.8	262.8
Number of streams	35	78	227	35	67	81	100	82	705
Drainage density	1.1	1.0	1.1	0.8	1.1	1.2	1.1	1.0	1.1
Stream frequency	3.1	3.5	2.9	2.6	2.4	3.4	2.5	2.8	2.9
Constant of channel maintenance	0.9	1.0	0.9	1.2	0.9	0.8	0.9	1.0	0.9
Drainage intensity	2.9	3.5	2.7	3.1	2.2	2.8	2.3	2.8	2.7
Infiltration number	3.3	3.5	3.2	2.1	2.6	4.1	2.7	2.9	3.1

The analysis of these parameters aims to gain insight into their physical attributes and their relationship with land degradation. Among the sub-basins, Mghougha is the largest in size, while Msaben is the smallest. Boukhalef stands out with the longest length, and Msaben has the shortest length. Mghougha exhibits also the highest perimeter. Most sub-basins have an elongation ratio close to 0.7, suggesting non-circular and compact shapes with irregular configurations. Concerning topography, Msaben and Spartel-Achkkar share the highest maximum altitude, while all sub-basins have a minimum altitude of 0 m. Msaben has the highest relief ratio, indicating significant altitude variation. Generally, the ruggedness of the sub-basins is low, implying relatively smooth terrains. In terms of drainage, Mghougha has the longest stream length, whereas Msaben has the shortest. Notably, Gueznaia exhibits the highest number of streams, contributing to a dense and complex hydrological network. The drainage density among sub-basins is relatively consistent. Msaben shows the highest stream frequency. The constant of channel maintenance values is consistent overall. These morphometric characteristics play a significant role in influencing the sub-basins' response to hydrometeorological flash events, leading to rapid and aggressive erosive processes.

The hydrological network in the sub-basins is dense and complex, with a high stream frequency due to the presence of several ridges. This results in a higher drainage intensity, indicating intense runoff and a stronger flow in the drainage network. The hydraulic response is rapid, mainly torrential with peaks closely related to precipitation, and the infiltration number suggests a more permeable soil that allows for greater infiltration of water.

These observations suggest that the sub-basins are vulnerable to land degradation, including small-scale geohazards such as flash floods, debris flows, shallow landslides, and slow extensive landslides, which can converge into a single large-scale disaster. The morphometric analysis provides valuable insights into the physical characteristics of the landforms in the sub-basins and helps to identify areas that are more prone to degradation and erosion.

**Figure 2:**
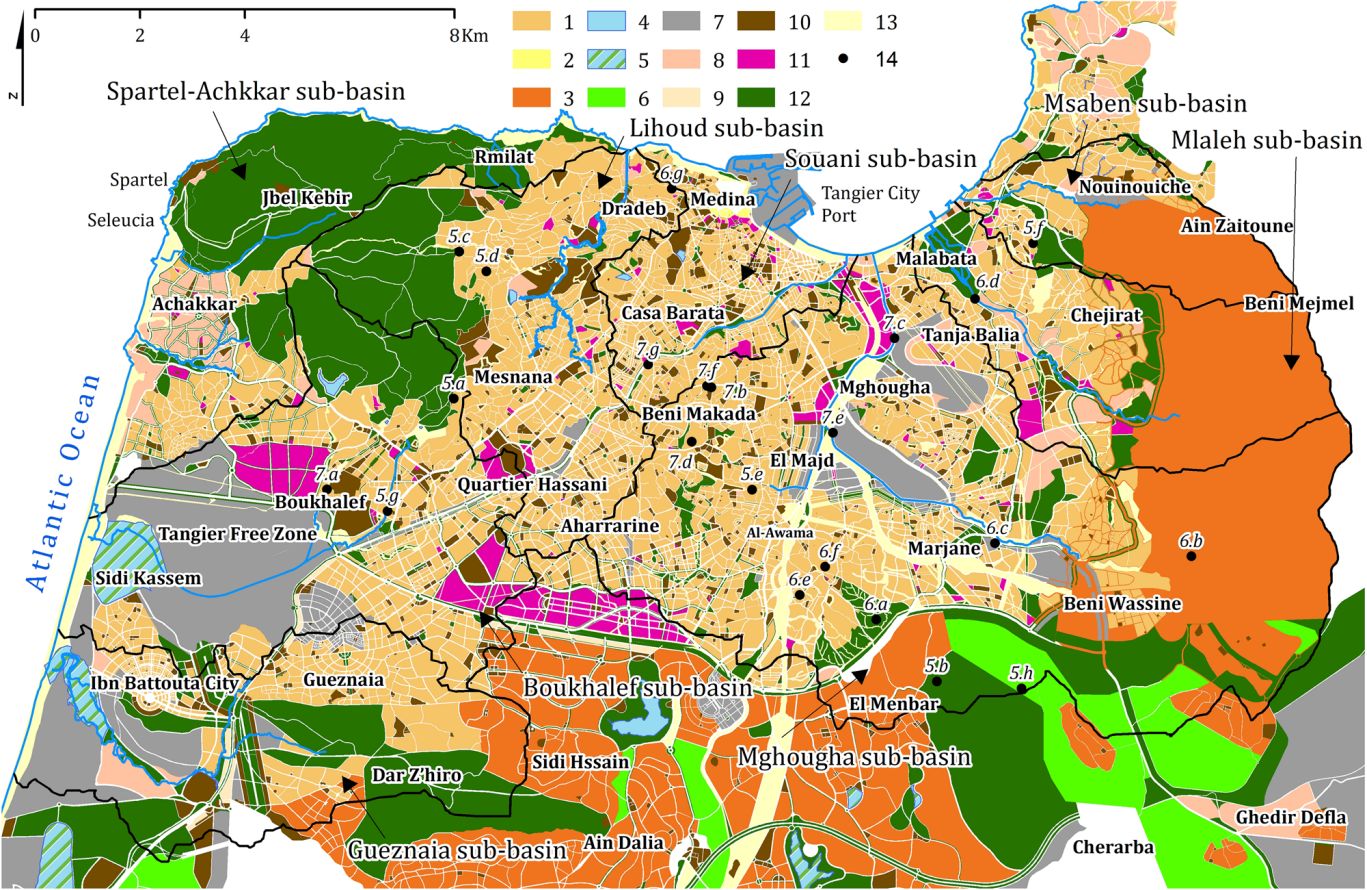
The requirements of the spatial configuration according to the designs in force in the study area: (1) urban housing area; (2) urban redevelopment area; (3) rural and peri-urban housing area; (4) water retention basins; (5) wetlands; (6) agricultural area; (7) areas of industrial and service activities; (8) tourist activity area; (9) commercial storefront area; (10) urban areas under special provisions; (11) urban expansion area; (12) vegetal landscape park; (13) other historical and natural landscapes; (14) position of shots in Figs. 5, 6 and 7 and their numbers (this figure was generated using ArcGIS 10.6 https://desktop.arcgis.com/en/arcmap/10.6/get-started/installation-guide/installing-on-your-computer.htm).

The spatial configuration according to the designs in force in the study area (Fig. 2) shows that the areas most prone to degradation are mainly associated with the steepest slopes surrounding the downtown area. In particular, the east, west, and south exhibit the greatest vulnerability, which corresponds to upstream of the sub-basins of Spartel-Achkkar, Mlaleh, Msaben, Mghougha and Gueznaia. These areas are characterized by uncontrolled urban and peri-urban extensions and are deficient in basic infrastructure, making them the most disadvantaged and marginalized areas in terms of access to essential services and facilities.

This certainly poses problems of priority allocation of preventive measures and planning control for three reasons: firstly, the structural fragility of these areas is heightened by constant anthropogenic pressure, increasing the risk of damage and instability. Secondly, the potential human and material damage resulting from unregulated and hasty expansion of construction, combined with the structural characteristics of these districts, is severe. Finally, the narrow morphology of the sub-basins and the short and narrow streams, along with sudden concentrated precipitation^16,27^, result in drainage systems unable to delay the arrival of flows to the low areas of the downtown. Consequently, strategic infrastructure along the course and downstream is at high risk of damage. Prioritizing the allocation of preventive measures and implementing town planning control measures is crucial to mitigate the risks posed by these factors, safeguard the structural integrity of vulnerable areas, and ensure the equitable and sustainable development of the city.

The morphology of the sub-basins significantly influences their response to hydrometeorological flash events, leading to rapid and aggressive erosive processes. The study area's complex nature, characterized by diverse topography and a dense hydrological network, makes it challenging to prioritize any specific sub-basin over others in terms of susceptibility to land degradation. Instead, the interconnectedness of these sub-basins necessitates an integrated management approach that considers all eight sub-basins collectively, rather than singling out individual ones. Given the multifaceted challenges and vulnerabilities observed across the study area, a comprehensive and holistic solution is necessary. This approach should encompass strategic infrastructure development, town planning control measures, and preventive actions to mitigate risks, ensure the structural integrity of vulnerable areas, and promote equitable and sustainable development within the metropolis.

### Spatial variability and drivers affecting soil erosion

**Figure 3:**
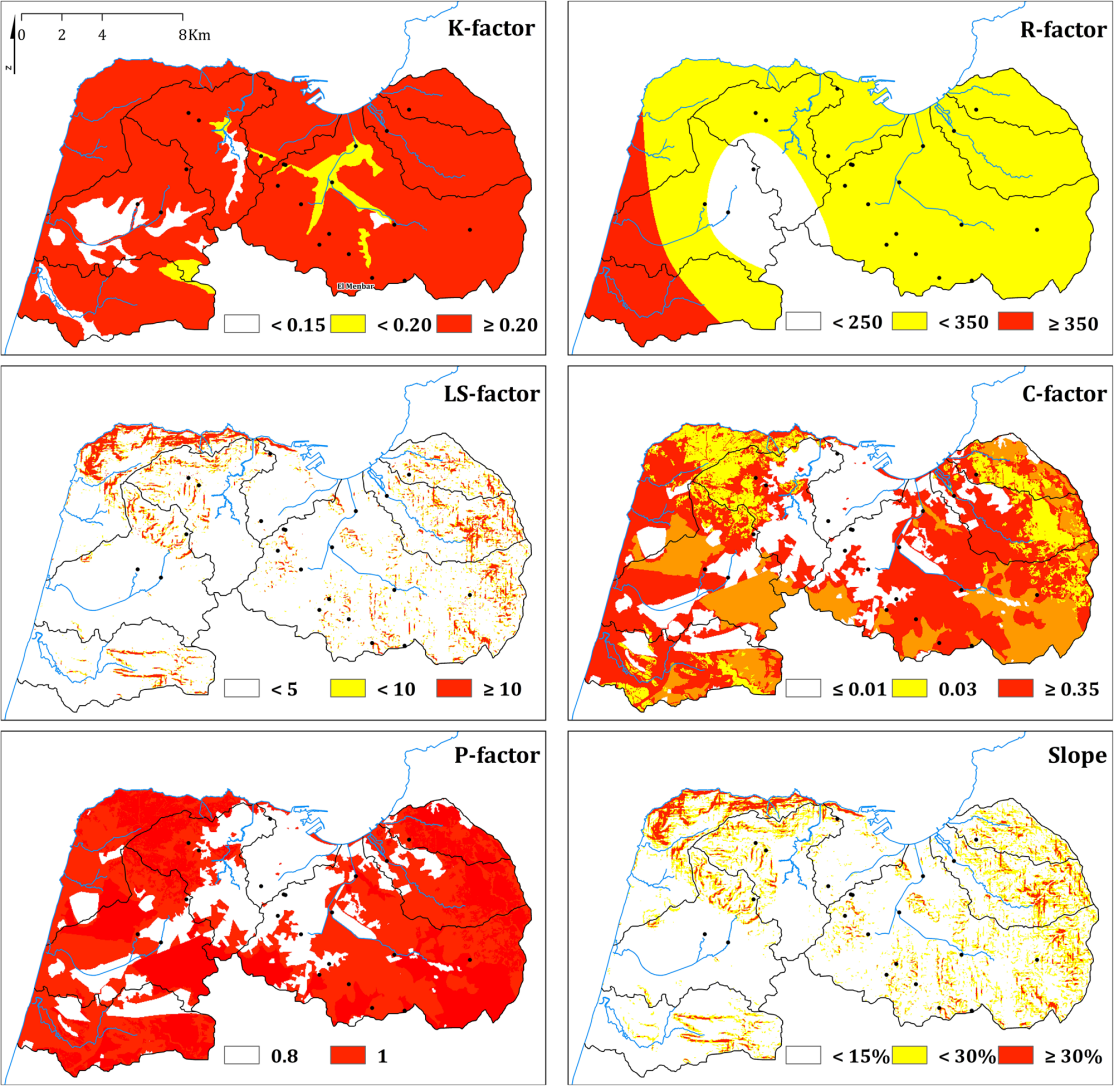
Spatial variability of RUSLE parametric maps (K, R, LS, C, and P) and slope in the study area, showcasing areas of high and low values using a simplified color-code (this figure was generated using ArcGIS 10.6 https://desktop.arcgis.com/en/arcmap/10.6/get-started/installation-guide/installing-on-your-computer.htm).

For parametric evaluation, the highest values (≥ 350) of the R-factor are found along the Atlantic coast (southwest) due to the arrival of wet ocean masses (Fig. 3). Apart from that, the entire center and west of the study area (88.1%) are dominated by values below 350. The mountainous block of Jbel El Kebir acts as a barrier, likely causing a relative reduction in rainfall aggressiveness towards the center and east due to the obstruction of wet oceanic masses.

The preponderance of the clayey and loamy facies makes values greater than 0.20 predominant for the K-factor (83.3%) (Fig. 3).

Most of the study area (86.5%) exhibits gentle slopes (Fig. 3), except for the eastern, northwestern, and southern regions, which experience higher slope values. Consequently, these areas have higher LS-factor values (≥ 10).

For the C-factor (Fig. 3), the built-up area (center and west) and water bodies (26.5%) receive the lowest values (≤ 0.01), forests (0.03) in the mountains to the northwest and northeast occupy 12.2% of the area, and higher values (≥ 0.35) correspond to widespread exposed soils (fallow) and agricultural lands (61.3%).

Finally, a few practices to reduce erosion such as linear and discontinued grassed waterways, linear erosion control structures, and small flood control ponds, resulted in high P-factor values (≥ 0.8).

**Figure 4:**
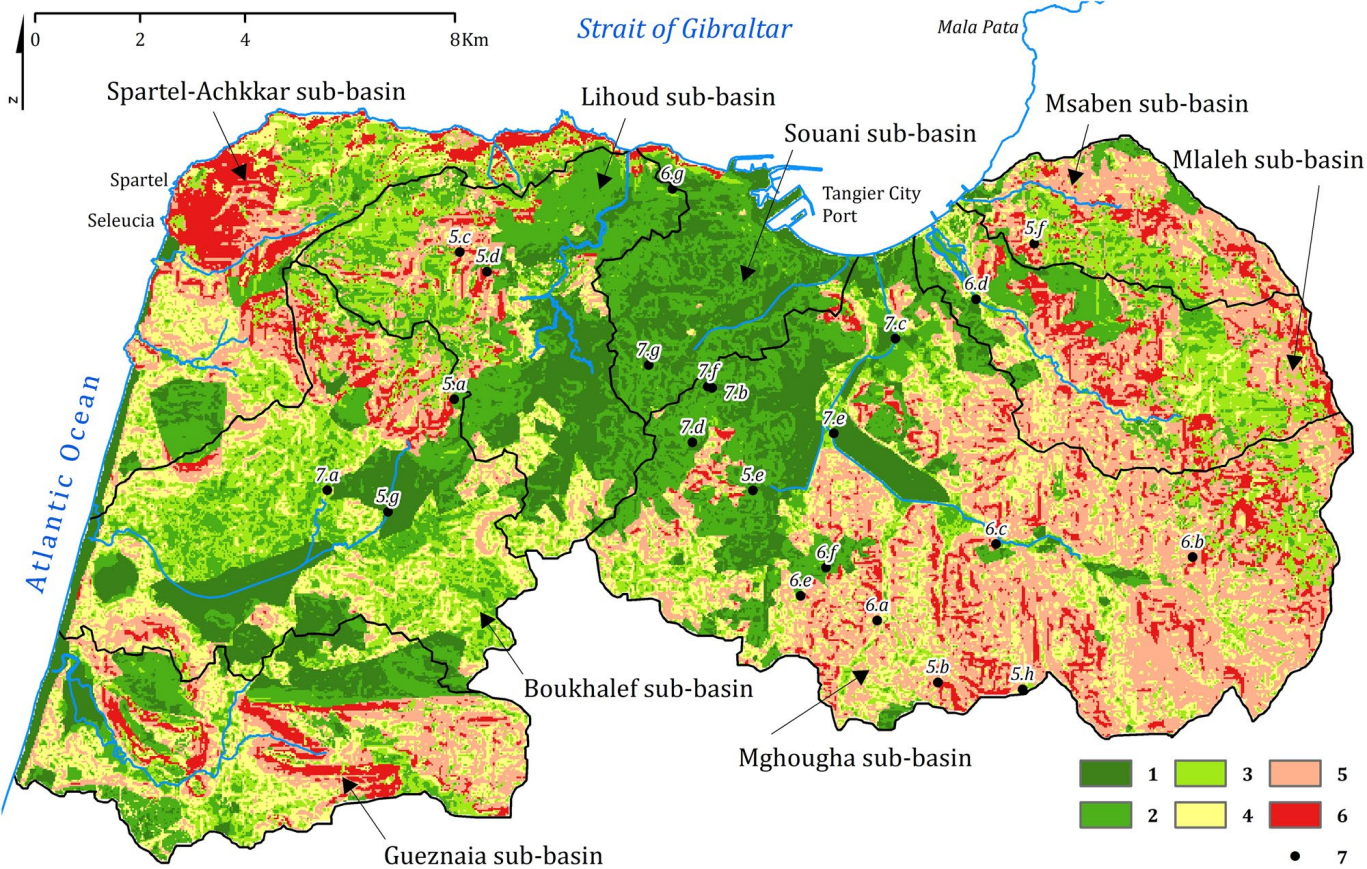
Soil loss map of the study area. Soil loss classes (t/ha/year): (1) ≤ 0.5; (2) [0.5, 5]; (3) [5, 15]; (4) [15, 50]; (5) [50, 200]; (6) > 200. (7) Position of shots in Figs. 5, 6 and 7 and their numbers (this figure was generated using ArcGIS 10.6 https://desktop.arcgis.com/en/arcmap/10.6/get-started/installation-guide/installing-on-your-computer.htm).

Soil loss has been divided into six classes (Fig. 4) according to an indicative color scale (from green to red)^45^. Three groups emerge:

- Low to very low erosion (green) with rates of less than 15 t/ha/year. It covers 124.4 km^2^ (51.1%) in the center and the west. The determining factors are LS (low slopes), C, and P.
- Medium erosion (yellow) with rates ranging up to 50 t/ha/year covers 42.5 km^2^ (i.e., 17.5%). This category often occurs in the hills and mountainsides, and the specific factors contributing to medium erosion can vary across different regions. In one hand, in the South (upstream Mghougha sub-basin), the factors K and R exhibit a monotonous pattern, indicating their lesser influence, while factors C, LS, and P play a more significant role in contributing to the medium erosion rates. On the other hand, in the South–West, the impact of the R-factor is highly relevant, making it the primary driver of erosion rates in combination with factors C, P, and K. These regional variations highlight the complex interplay of multiple factors in shaping medium erosion patterns.
- High to very high erosion (orange and red) with rates above 50 t/ha/year covering 76.34 km^2^ (i.e., 31.4%). They are observed in the mountains (west, east, and south) as an outcome of the combination of the high soil erodibility (C-factor), the absence or failure of conservation measures (P-factor), and the steep slopes (LS-factor). The effect of more significant rainfall erosivity (R factor) is mainly observed in the northwest.

In conclusion, land degradation in our case is influenced by multiple factors contributing to erosive processes, including friable lithology, poor soils, and steep slopes combined with a lack of conservation measures.

The average potential soil erosion rate is 24.2 t/ha/year which corresponds to a total annual soil loss of 588,051 t/year. This high value approaches the average regional rate of 27.7 t/ha/year^46–48^. It is 99.8% caused by the disastrous erosion risk category which covers 8.3% of the territory. The most vulnerable areas are recently burned topsoil and fallow land combined with steep slopes located in several places to the east, west, and south. These places are home to both uncontrolled neighborhoods and areas of planned urban and industrial expansion, which compromises the sustainability of the landscape and the socio-economic prospects. Moreover, steep slopes (LS-factor) and rainfall erosivity (R-factor) appear to be the predominant factors that accentuate the effect of other factors for the detachment and transport of particles and debris.

This results in spatially varying erosion rates, which are quite a high^46,49–50^. The spatial association between the findings of the soil loss assessment and the morphometric analysis consolidates the conclusions drawn regarding the damage and its consequences. The following section will focus on field monitoring, which will be used for validation and further assessment.

### Field assessment and validation

**Figure 5:**
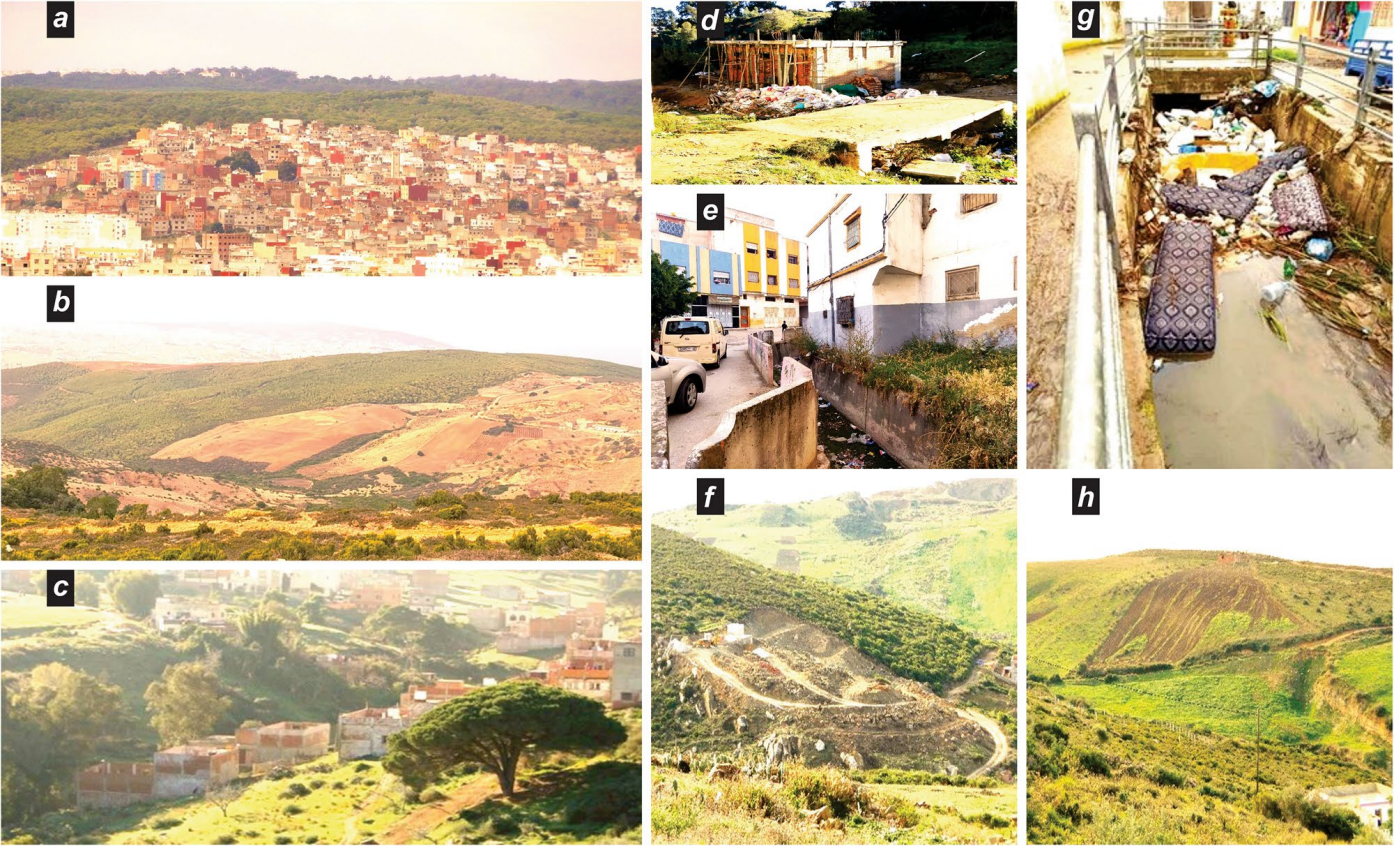
Anthropogenic drivers contributing to erosion vulnerability and impacts in the study area. (**a**) Urban sprawl in place of forests on slopes; (**b**) expansion of agriculture on forested slopes; (**c**) building on creek beds during dry periods; (**d**) construction in stream banks and beds; (**e**) construction pressure on drainage channels leading to flooding, destruction, and water leakage, (**f**) start of a construction project on the slopes and deforestation; (**g**) waste blocking waterways; (**h**) upstream plowing in the direction of slopes. Credits attribution: pictures from the authors.

The field assessment validates the findings of the modeling spatially and shows that the erosive processes reinvigorate floods by discharging a large sediment load, which is deposited in valleys and causes significant damage to infrastructure, equipment, and soil. Moreover, there are many anthropogenic actions that are precursors to land degradation, particularly upstream in peri-urban and urban expansion areas (Fig. 5).

Field monitoring and remote sensing analysis have provided valuable insights into the hydroclimatic hazards within the study region. Severe flash floods have been observed in the upstream areas of the western sub-basins (such as Msaben, Mlaleh, and Mghougha), as well as in the mountainous regions of Jbel Kebir (northwest) and Dar Z'hiro (south), known for their high sediment loads^5,16^. In contrast, downstream areas are more susceptible to flooding. These findings contribute to our understanding of the specific types of hazards present in each area, highlighting the importance of considering local topography and sediment dynamics in assessing hydroclimatic risks. Furthermore, intense storms' impact on the already friable soils and steep slopes is characterized by violent concentrated precipitations and torrents. These factors make the study area highly susceptible to soil detachment and erosion^16,25,27^. Moreover, the influence of long drought periods leads to reduced vegetation cover and soil moisture, further increasing the vulnerability of the soil to erosion during subsequent rainfall events^26,47,51^. These specific impacts of storms and long droughts on the region's friable soils and steep slopes highlights the urgent need for proactive measures to mitigate these hydroclimatic conditions' adverse effects^51–55^. Implementing conservation practices, improving land management strategies, and employing soil stabilization techniques are crucial steps in protecting the soils from erosion and preserving their quality.

**Figure 6:**
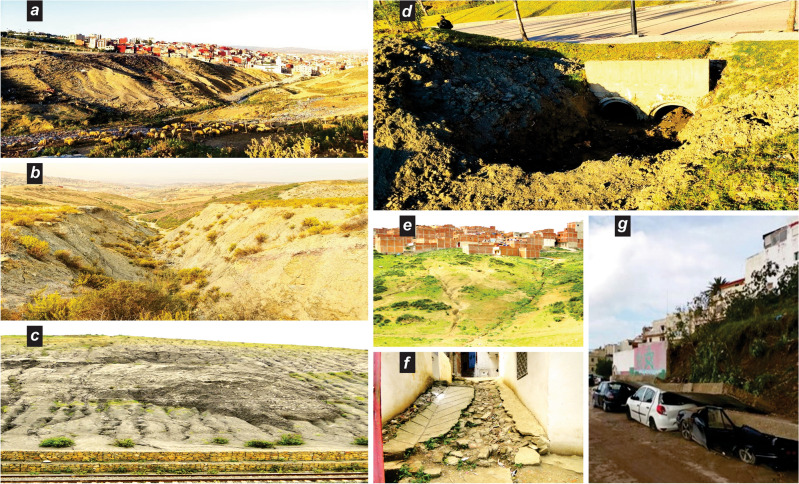
Inadequate upstream measures leading to drainage channel blockages and examples of consequent damage. (**a**) Muds and debris clogging drainage in a waterway in an outlying neighborhood; (**b**) erosion canyon overlooking hydrographic axis flowing towards industrial zone and Mghougha depression flood barrier; (**c**) mass landslide upstream of the Mghougha sub-basin; (**d**) complete obstruction of essential drainage channels; (**e**) evolution of erosion shapes in the vicinity of upstream semi-urban areas; (**f**) damage to alleyways and public walkways turned into natural drainage channels for torrents; (**g**) damage to properties resulting from land degradation. Credits attribution: pictures from the authors.

The steep slopes, lack of vegetation cover in the upstream sub-basins, and the presence of friable soil make the area vulnerable to various forms of erosion such as rills, gullies, and cut banks. These erosion features are abundant on the slopes surrounding the downtown, leaving behind deposits like stream terraces and alluvial fans on and near the slopes, as well as in watercourses and low-lying areas (Fig. 6). The absence and/or non-compliance of preventive measures leads to blockages of drainage channels and causes severe and sequential human and material damage.

**Figure 7:**
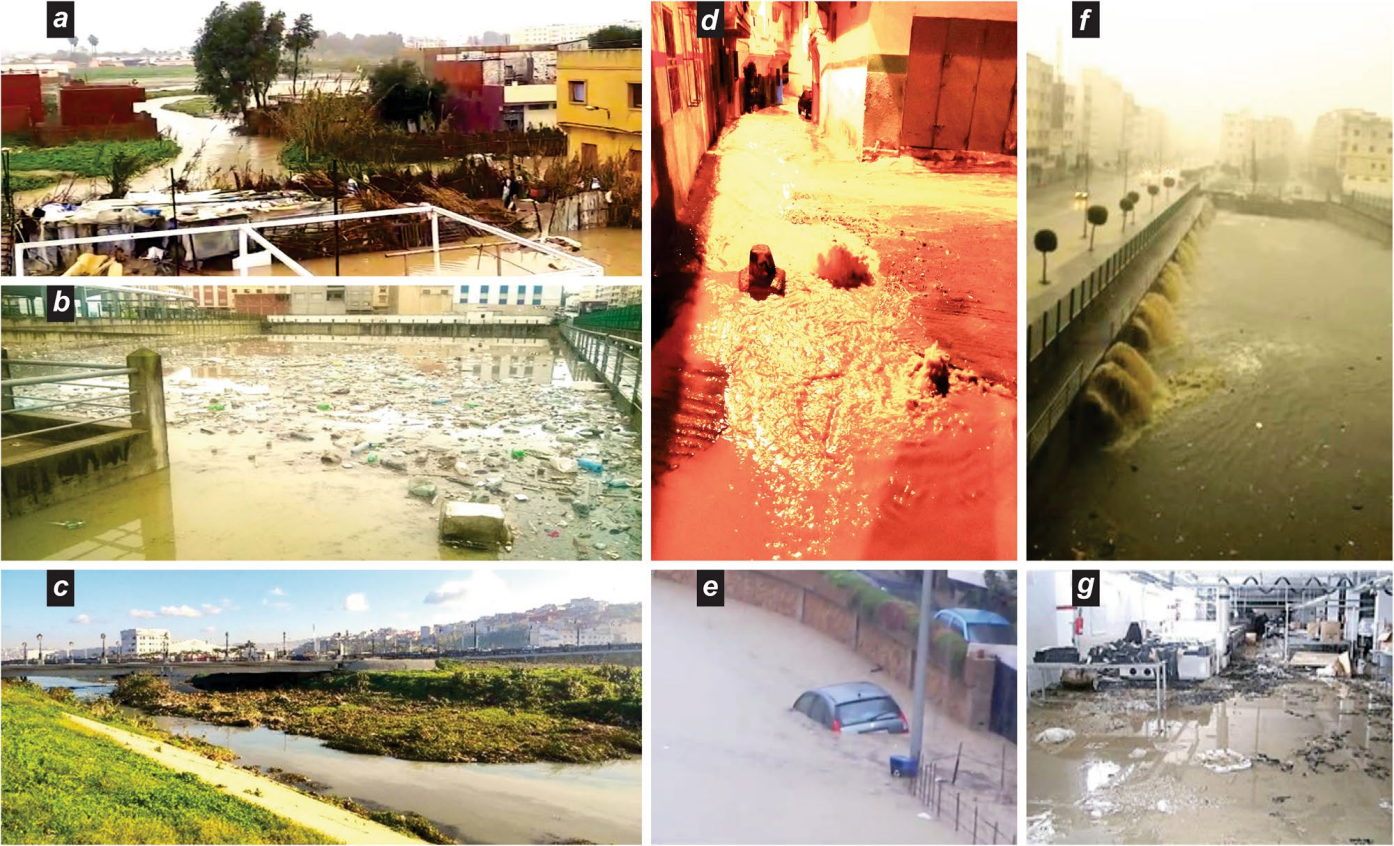
The effects of inadequate water management in downstream neighborhoods and industrial zones. (**a**) Unsafe building practices in water courses lead to disastrous effects of erosion and flooding; (**b**) lack of maintenance hinders the effectiveness of flood water storage tanks in protecting neighborhoods; (**c**) sediment build-up impairs winter water drainage and alters the water flow path with devastating results; (**d**) ineffective winter water containment and drainage causes artesian rise and damage to tiled alleys and housing; (**e**) high valley water level causes drowning of cars and damages in the industrial zone of Mghougha; (**f**) torrential rains with high solid load impact roads downstream; (**g**) high sedimentary load causes loss of lives, damage to factory machinery, and production halts with exacerbated losses. Credits attribution: pictures from the authors.

This leads to environmental and development impacts that threaten biodiversity and water resources, which are essential for ensuring nutrition and stability. For example, a shrinking of coastal lagoons and marshes (e.g., Sidi Kassem) is observed due to development works and construction projects. Similarly, frequent high sediment deposits are observed (Fig. 6a,d, 7c,g) in water canals and reservoirs (e.g., irrigation dams on the hillsides of the Boukhalef Plain) due to inadequate protection measures (Figs. 5c–g, 7a,b,d). Agricultural activity is still important in peri-urban areas, providing job opportunities and supplying local markets. However, the consequences of erosion, such as the deformation of fields and land retreat (e.g., gullies that reduce arable land), result in a decline in agricultural activity.

It is essential to acknowledge that the assessment of economic losses resulting from land degradation in the specific industrial zones in downstream, such as the former free zone of the port of Tangier City, the Tangier free zone, and the industrial zones of Mghougha and Al-Awama, is based on information available from media reports and anecdotal evidence. While there is a lack of official data on the extent of economic losses caused by erosion and flooding hazards in these areas, the media coverage and news reports provide insights into the significant damages experienced by various stakeholders^56–60^.

For instance, during recurrent annual floods (e.g., 2008, 2009, 2021, 2022, and 2023), the floodwaters carrying sediment loads can reach heights of up to three meters due to intense precipitation and poor rainwater evacuation. These flood events, characterized by high sediment loads, substantially damage machinery, manufacturing workshops, and storage warehouses^61,62^ (Fig. 7e–g). Infrastructure elements like roads, alleys, public passages, and drainage channels are also affected, particularly in poorly equipped neighborhoods (Fig. 7a–f). These damages, including overturned electric wire poles and choked wastewater drainage networks, only disrupt the natural hydrological dynamics and pose risks to the safety of homes and lives. The social effects of such events are profound, resulting in psychological distress among the affected population and leading to recurring resentment and protest. Furthermore, these disturbances to the natural hydrological processes hinder coastal enrichment and impact the capacity to renew coastal dunes^63^. While official data specifically quantifying the economic losses is not available, the observed damages and their implications for the industrial zones and the overall socio-economic well-being of the area underline the relevance of considering and addressing the economic consequences of erosion and flooding hazards in Tangier.

In light of recent climatic constraints, urgent development needs, and significant risks posed by recurring damages^5,7,64,65^, the research findings emphasize the identification of structural weaknesses and consider it crucial to prioritize the hydraulic modeling of drainage channels to ensure proper and implementation of appropriate and cost-effective measures at the earliest prospect.

## Conclusions

This study investigated soil erosion and hydroclimatic hazards in Tangier’s major African port city. By employing morphometric analysis, soil loss calculation, field monitoring, and remote sensing and GIS tools, the extent of soil erosion was assessed. The findings highlight the urgent need for effective land management practices and conservation measures to mitigate the impacts of land degradation and soil erosion, particularly in rapidly developing urban areas. The use of remote sensing and GIS technologies provided valuable insights into the physical characteristics and vulnerability of the Tangier Metropolis to these hazards.

The results showed an average soil erosion rate of 24.2 t/ha/year, resulting in an annual soil loss of 588,051 t/year. This high rate can be attributed to areas with a high erosion risk, which cover only 8.3% of the territory but account for 99.8% of the soil loss. These vulnerable areas, characterized by recently burned topsoil, fallow land, and steep slopes, are found in both uncontrolled neighborhoods and areas designated for planned urban and industrial expansion. This poses a significant threat to the sustainability of the landscape and socio-economic prospects.

The spatial distribution of erosion rates is influenced by factors such as slope steepness, rainfall erosivity, soil erodibility, and the absence of conservation measures. The morphometric analysis further revealed that the eastern and western regions of Tangier are particularly susceptible to soil erosion, indicating the need for targeted conservation efforts in these areas.

Field monitoring validated the modelling findings and highlighted the link between erosive processes and floods, which deposit large sediment loads in valleys, causing infrastructure damage. Anthropogenic actions, particularly in upstream peri-urban and urban expansion areas, contribute to land degradation. The negative consequences of erosion, such as the deformation of fields and land retreat, have led to a decline in agricultural activity. Furthermore, Recurrent floods have caused significant damage to industry and infrastructure. This damage disrupts natural hydrological dynamics and pose risks to the safety of homes and lives with profound socioeconomic effects.

The results provide valuable information for decision-makers and stakeholders to develop strategies to mitigate the impacts of land degradation and enhance the durability and efficiency of spatial rectification. The latter, by aiming for durability and efficiency, must put an end to the anticipation of uncontrolled construction which, afterwards, makes any planning unrealistic and outdated. Certainly, further research and data collection are required to quantify economic losses accurately and assess the long-term effectiveness of conservation measures.

Halting the anticipation of uncontrolled construction is crucial as it prevents subsequent planning from becoming unrealistic and outdated. Obviously, this is an issue not exclusive to Tangier, it applies to fast-growing emerging cities in the Mediterranean and around the world, which face similar challenges and experience comparable impacts.

## Data Availability

Sentinel 2 and Landsat series datasets are available at https://eos.com/landviewer/, the Fertimap dataset is available at http://www.fertimap.ma/, SRTM v3 is available at https://www.earthdata.nasa.gov/.
